# Predicting response to checkpoint inhibitors in melanoma beyond PD-L1 and mutational burden

**DOI:** 10.1186/s40425-018-0344-8

**Published:** 2018-05-09

**Authors:** Carl Morrison, Sarabjot Pabla, Jeffrey M. Conroy, Mary K. Nesline, Sean T. Glenn, Devin Dressman, Antonios Papanicolau-Sengos, Blake Burgher, Jonathan Andreas, Vincent Giamo, Moachun Qin, Yirong Wang, Felicia L. Lenzo, Angela Omilian, Wiam Bshara, Matthew Zibelman, Pooja Ghatalia, Konstantin Dragnev, Keisuke Shirai, Katherine G. Madden, Laura J. Tafe, Neel Shah, Deepa Kasuganti, Luis de la Cruz-Merino, Isabel Araujo, Yvonne Saenger, Margaret Bogardus, Miguel Villalona-Calero, Zuanel Diaz, Roger Day, Marcia Eisenberg, Steven M. Anderson, Igor Puzanov, Lorenzo Galluzzi, Mark Gardner, Marc S. Ernstoff

**Affiliations:** 1 Center for Personalized Medicine, Roswell Park Comprehensive Cancer Center, Buffalo, NY 14263 USA; 2 Department of Pathology, Roswell Park Comprehensive Cancer Center, Buffalo, NY 14263 USA; 3 Cancer Genetics and Genomics, Roswell Park Comprehensive Cancer Center, Buffalo, NY 14263 USA; 4 OmniSeq Inc., Buffalo, NY 14203 USA; 5 Department of Hematology/Oncology, Fox Chase Cancer Center, Philadelphia, PA 19111 USA; 6 Department of Hematology and Oncology, Dartmouth Hitchcock, Lebanon, NH 03756 USA; 7 Department of Pathology, Dartmouth Hitchcock, Lebanon, NH 03756 USA; 8 Department of Pathology, Community Hospital, Munster, IN 46321 USA; 9 Department of Clinical Oncology Development, Hospital Universitario Virgen Macarena, 41009 Sevilla, Spain; 10 Department of Medicine, Columbia University, New York, NY 10032 USA; 11 Miami Cancer Institute, Baptist Health South Florida, Miami, FL 33176 USA; 12 Department of Biomedical Informatics and Biostatistics, University of Pittsburgh, Pittsburgh, PA 15213 USA; 13 Laboratory Corporation of America Holdings, Burlington, NC 27215 USA; 14 Department of Medicine, Roswell Park Comprehensive Cancer Center, Buffalo, NY 14263 USA; 15 Department of Radiation Oncology, Weill Cornell Medical College, New York, NY 10065 USA; 16 Sandra and Edward Meyer Cancer Center, New York, NY 10065 USA; 17 Université Paris Descartes/Paris V, 75006 Paris, France

**Keywords:** Pembrolizumab, Nivolumab, Ipilimumab, Algorithmic analysis, Inflamed, Borderline, Immune Desert

## Abstract

**Background:**

Immune checkpoint inhibitors (ICIs) have changed the clinical management of melanoma. However, not all patients respond, and current biomarkers including PD-L1 and mutational burden show incomplete predictive performance. The clinical validity and utility of complex biomarkers have not been studied in melanoma.

**Methods:**

Cutaneous metastatic melanoma patients at eight institutions were evaluated for PD-L1 expression, CD8^+^ T-cell infiltration pattern, mutational burden, and 394 immune transcript expression. PD-L1 IHC and mutational burden were assessed for association with overall survival (OS) in 94 patients treated prior to ICI approval by the FDA (historical-controls), and in 137 patients treated with ICIs. Unsupervised analysis revealed distinct immune-clusters with separate response rates. This comprehensive immune profiling data were then integrated to generate a continuous Response Score (RS) based upon response criteria (RECIST v.1.1). RS was developed using a single institution training cohort (*n* = 48) and subsequently tested in a separate eight institution validation cohort (*n* = 29) to mimic a real-world clinical scenario.

**Results:**

PD-L1 positivity ≥1% correlated with response and OS in ICI-treated patients, but demonstrated limited predictive performance. High mutational burden was associated with response in ICI-treated patients, but not with OS. Comprehensive immune profiling using RS demonstrated higher sensitivity (72.2%) compared to PD-L1 IHC (34.25%) and tumor mutational burden (32.5%), but with similar specificity.

**Conclusions:**

In this study, the response score derived from comprehensive immune profiling in a limited melanoma cohort showed improved predictive performance as compared to PD-L1 IHC and tumor mutational burden.

**Electronic supplementary material:**

The online version of this article (10.1186/s40425-018-0344-8) contains supplementary material, which is available to authorized users.

## Background

The introduction of immune checkpoint inhibitors (ICIs) revolutionized the clinical management of patients with metastatic melanoma [1–8]. However, only a minority of patients obtain durable clinical benefit from ICIs. [1, 4, 6–8] Moreover, ICI-based immunotherapy is associated with significant immune related side effects [1, 4, 6–8], and it has an estimated cost of over $300,000 per quality-adjusted life-year, [9] calling for the development of robust predictive biomarkers [10]. Despite considerable efforts in this direction, the identification and validation of biomarkers that would predict the response of melanoma patients to ICIs targeting cytotoxic T lymphocyte associated protein 4 (CTLA4) or programmed cell death 1 (PDCD1, best known as PD-1), has not been very successful so far. In particular, neither assessing intratumoral expression levels of CD274 (best known as PD-L1) by immunohistochemistry, nor evaluating tumor mutational burden by whole-exome sequencing appears to suffice to predict long-term clinical benefits [3–8, 10–13]. Moreover, it has become clear that robust predictions may involve different biomarkers in patients receiving CTLA- versus PD-1-targeting agents [4, 14]. The results of the CHECKMATE 067 clinical trial demonstrated that better objective response rates (ORRs) and overall survival (OS) in PD-L1 negative patients for the combination of dual agent CTLA-4 and PD-1 targeting ICIs [14, 15]. Based on these findings, some oncologists are currently using negative PD-L1 expression to move past monotherapy and prescribe dual ICIs, while others consider patient preference and perceived tolerability without biomarkers to aid clinical decision making, despite the risk of increased rates of adverse events (AEs) [14]. A global investigation of the mutational and immunological aspects of the disease, going beyond the standalone assessment of mutational burden and PD-L1 expression levels, is critical to the development of superior predictive biomarkers for melanoma patients treated with ICIs.

The genomic landscape of melanoma, performed by The Cancer Genome Atlas (TCGA) on 333 melanoma samples delineated 4 genomic subtypes, i.e., triple wild-type (WT), BRAF mutant, NRAS, HRAS or KRAS mutant, and NF1 mutant, as well as three transcriptomic subclasses, i.e., immune, keratin and MIFT-low. [16] Although this provided several useful insights into the disease, it did not improve our ability to predict clinical responses to ICIs amongst melanoma patients. To address this gap, we performed a comprehensive analysis of the mutational and immunological landscape of 300 samples from metastatic melanoma patients at eight institutions worldwide using a CLIA/CAP-certified NYS-approved laboratory developed test (LDT) [17]. This test evaluates PD-L1 expression levels, CD8^+^ T-cell tumor infiltration pattern by immunohistochemistry, mutational burden by whole-exon DNA-seq, as well as the abundance of 394 immune transcripts by RNA-seq. [17] Each of these parameters was studied individually in a dichotomous manner for their influence on ORR and OS in patients undergoing surgery prior to the approval of ipilimumab in 2011, i.e. historical controls, versus patients receiving ICI-based immunotherapy, which allowed for the differentiation between prognostic and predictive biomarkers. Finally, a training cohort of 48 ICI-treated patients was used to develop a continuous response score (RS), subsequently tested on a validation cohort of an additional 29 ICI-treated patients.

## Methods

### Patients and clinical data

Eight collaborating institutions obtained approval by their respective institutional review boards (IRBs) to submit existing de-identified specimens and associated clinical data for use in this study (Additional file 1: Table S1). A total of 300 patients were included in the study, based on the following criteria: history of metastatic cutaneous melanoma with surgical resection of a primary or metastatic tumor; availability of adequate archival formalin-fixed paraffin-embedded (FFPE) tissue collected prior to treatment with ICIs; availability of sequencing data; and availability of demographic, diagnosis, follow-up and vital status data. Patients were excluded if they died within three months of initial diagnosis, or were alive at last follow-up, but had less than six months of follow-up time from initial diagnosis of metastatic disease. Based on the initial approval of ipilimumab by the US FDA (March 25th, 2011), patients with biopsies demonstrating Stage IV disease prior to the availability of ICIs (1992–2010) were included as historical controls (*n* = 94). Medical and electronic pharmacy records were reviewed to identify individuals receiving FDA-approved checkpoint inhibitors (*n* = 166) for which a subset (*n* = 137) with a median follow-up of 16.2 months was used for survival analysis. This latter group of 137 patients is referred to as the ICI-treated group (Table 1), and for which 78 of these were evaluable and 59 not evaluable (Additional file 1: Table S8) for response to treatment by RECIST v1.1 (Additional file 1: Table S1) [18]. From this group of 78 patients a total of 77 had complete measurements and were divided into a training set (48 patients) from a single institution (RPCCC) and a validation set (29 patients) from eight different institutions and were used to develop a Response Score (RS) for prediction of response to ICI. The training cohort represented the largest population from a single institution, whereas the validation cohort represented multiple patients from various institutions. A complete review of all patient dispositions is included in the Additional file 7 (Additional file 2: Figure S1).

**Table 1:** Clinical characteristics

	All Cases^a^	Pre-ipi approval	Post-ipi approval	Post-ipi approval	
	(*n* = 300)	(*n* = 94)	(*n* = 206)	(*n* = 206)	
				ICI Treated	ICI Not Treated
Age at initial cutaneous melanoma diagnosis (Years, %)				(*n* = 160)	(*n* = 46)
< 30	9 (03.0)	5 (05.3)	4 (01.9)	2 (01.3)	2 (04.3)
30–39	25 (08.3)	11 (11.7)	14 (06.8)	9 (05.6)	5 (10.9)
40–49	49 (16.3)	15 (16.0)	34 (16.5)	26 (16.3)	8 (17.4)
50–59	69 (23.0)	25 (26.6)	44 (21.4)	37 (23.1)	7 (15.2)
60–69	68 (22.7)	17 (18.1)	51 (24.8)	40 (25.0)	11 (23.9)
70–79	51 (17.0)	13 (13.8)	38 (18.4)	31 (19.4)	7 (15.2)
≥ 80	29 (09.7)	8 (08.5)	21 (10.2)	15 (09.4)	6 (13.0)
Mean	59	56	60	61	58
Year of diagnosis (Range)	1974–2016	1974–2010	1989–2016	1990–2016	1989–2016
Sex					
Female	111 (37.0)	36 (38.3)	75 (36.4)	53 (33.1)	22 (47.8)
Male	189 (63.0)	58 (61.7)	131 (63.6)	107 (66.9)	24 (52.2)
Race					
White	293 (97.7)	94 (100.0)	199 (96.6)	154 (96.3)	45 (97.8)
Other	2 (06.0)	0 (00.0)	2 (01.0)	1 (00.6)	1 (02.2)
Unknown	5 (01.7)	0 (00.0)	5 (02.4)	5 (03.1)	0 (00.0)
Vital status at last follow up					
Alive	136 (45.3)	16 (17.0)	120 (58.3)	92 (57.5)	28 (60.9)
Dead	164 (54.7)	78 (83.0)	86 (41.7)	68 (42.5)	18 (39.1)
Year of bx proven Stage IV disease (range)	1992–2017	1992–2011	2011–2017	2011–2017	2011–2017
Months of follow up (Median)^b^	16.2	16.5	16.2	16.2	15.5
< 1	13 (04.3)	0 (00.0)	13 (06.3)	10 (06.3)	3 (06.5)
3	122 (40.7)	0 (00.0)	122 (59.2)	104 (65.0)	18 (39.1)
6	74 (24.7)	3 (03.2)	71 (34.5)	46 (28.8)	25 (54.3)
10	42 (14.0)	42 (44.7)	0 (00.0)	0 (00.0)	0 (00.0)
> 10	49 (16.3)	49 (52.1)	0 (00.0)	0 (00.0)	0 (00.0)
Median	4.4	10	3	2.8	4.2
Years from diagnosis to bx proven Stage IV disease (Median)	1.1	1.5	1	1	0.7
Received BRAF TKI (Yes, %)	25 (08.3)	0 (00.0)	25 (12.1)	19 (11.9)	6 (13.0)
Mo from specimen collection to BRAF TKI (Median)	6·5	N/A	6·5	8	6
Checkpoint inhibitor					
Ipilimumab				72 (45.0)	N/A
Pembrolizumab				68 (42.5)	N/A
Nivolumab				7 (04.4)	N/A
Ipilimumab + Nivolumab				13 (08.1)	N/A
Time to progression (median days)				77.5	N/A
Progression free survival (median days)				129.5	N/A

^a^All cases are metastatic and no cutaneous samples included. ^b^For pre-ipi approval patients follow-up is the number of months from date of specimen collection (bx proven Stage IV disease) to last date of follow up or date of death, and for post-ipi approval patients represents the number of months from date of first dose of checkpoint inhibitor to last date of follow up or date of death

### Immunohistochemical studies

The expression of PD-L1 on the surface of tumor cells was assessed in all samples by means of the Dako Omnis platform (Agilent, Santa Clara, CA) and the 28–8 pharmDx antibody. Expression levels, and were scored as per published guidelines [19]. Additional serially sectioned tissue was evaluated for lymphocyte infiltration using the anti-CD8 antibody C8/144B (Agilent, Santa Clara, CA) and assigned a qualitative score of non-infiltrated, infiltrated, or excluded. Non-infiltrated referred to a sparse number of CD8^+^ T-cells that infiltrate nests of neoplastic cells and with less than 5% of the tumor showing an infiltrating pattern. Infiltrated represents frequent CD8^+^ T-cells that infiltrate nests of neoplastic cells in an overlapping fashion at least focally and in more than 5% of the tumor. Excluded represents restriction of more than 95% of all CD8^+^ T-cells in a tumor to the periphery or interstitial stromal areas and not actively invading nest or groups of neoplastic cells.

### Mutational burden and RNAseq profiling

DNA and RNA was co-extracted from each sample and processed for whole-exon DNAseq or RNAseq as previously described [17, 20]. Mutational burden and gene expression were evaluated by targeted capture and sequencing of 409 cancer-related genes and amplicon sequencing of 394 immune transcripts, respectively, on samples that met validated quality control (QC) thresholds [17]. Somatic mutation calling was conducted using Ion Torrent Suite software’s variant caller plugin (for detailed information, please refer to Additional file 7). Mutational burden cutoff was derived from a reference population whereby the median MuB was 3.55 mutations per megabase DNA. This value was used as a baseline and a high MuB was defined as 2× this median value (7.1).

### Data analyses

Survival analysis was performed using a log-rank test on 5-year Kaplan-Meier survival curves for immune markers including PD-L1 by IHC, pattern of CD8 expression by IHC, mutational burden by DNA-Seq and targeted transcriptome profiling of 394 immune-related genes by RNA-Seq (for detailed information, please refer to Additional file 7). Hierarchical clustering with Pearson’s correlation dissimilarity was performed on 394 expression ranks for all samples. Overrepresentation test was performed on clusters to determine gene enrichment, mutational landscape, other primary biomarkers and clinical characteristics. A relative likelihood of response to ICI, referred to as the response score (RS), was calculated based upon a weighted score of a generalized linear model with additional input from CD8 expression and CD8^+^ T-cell infiltration pattern. The RS was developed on 48 training patients and tested on a validation set of 29 patients.

## Results

### PD-L1 immunohistochemistry and mutational burden

**Fig. 1:**
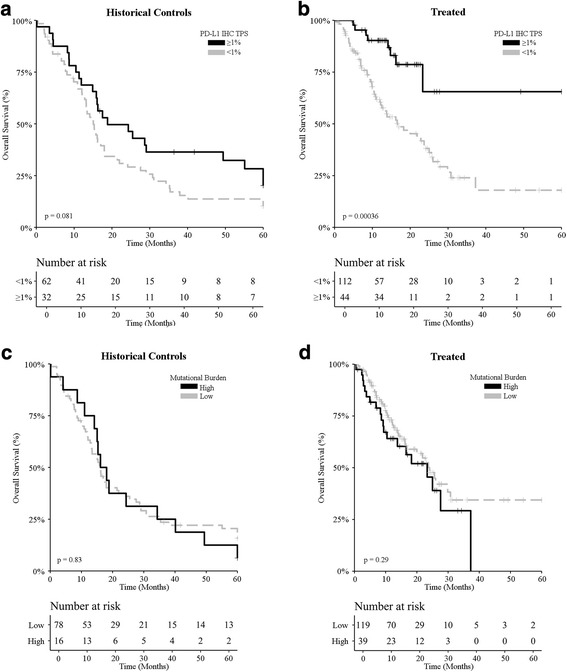
Survival analysis of melanoma patients based on PD-L1 expression levels and mutational burden. **a**, **b** Overall survival upon stratification based on PD-L1 positivity (tumor proportion score ≥ 1% versus < 1%) for metastatic melanoma patients treated (**a**) prior to the introduction of ICIs (historical controls; *n* = 94) and (**b**) ICI-treated melanoma patients (*n* = 137). **c**, **d** Overall survival upon stratification based on mutational burden [high (≥ 7.1 mut/Mb) versus non-high] for metastatic melanoma patients treated (**c**) prior to the introduction of ICIs (historical controls; n = 94) and (**d**) ICI-treated melanoma patients (n = 137). *p*-values are indicated

PD-L1 positivity (> 1% tumor cells with membranous PD-L1 staining) [14, 15] was documented in 33% (98/298) of all samples compatible with immunohistochemical evaluation. Amongst ICI-treated patients, PD-L1 positivity was associated with 55.6% ORR, while only 37.9% of patients bearing PD-L1-negative melanoma achieved an objective clinical response upon ICI-based immunotherapy (Additional file 1: Table S3). ORR for higher cut-off values for PD-L1 IHC showed similar results (Additional file 1: Table S7). A trend for improved OS was observed amongst patients bearing PD-L1-positive melanomas and undergoing treatment before the introduction of ICIs (Fig. 1a; *p* = 0.081; Additional file 1: Table S2). However, the impact of PD-L1 positivity on OS was much more considerable amongst patients receiving ICI-based immunotherapy (Fig. 1b; *p* = 0.00036; Additional file 1: Table S2), corroborating the predictive value of PD-L1 expression levels measured by IHC and stratified based on a 1% cut-off value.

A high mutational burden [3, 21], more than 7.1 somatic nonsynonymous mutations per million exonic bases, could be documented in 21% (63/300) of all patients. High mutational burden was associated with 33% ORR amongst ICI-treated patients, while only 21% of patients with a melanoma characterized by comparatively lower mutational burden achieved an objective clinical response following ICI-based immunotherapy (Additional file 1: Table S3). Mutational burden had no statistically significant impact on OS, neither amongst historical controls (Fig. 1c; *p* = 0.83; Additional file 1: Table S2), nor amongst ICI-treated patients (Fig. 1d; *p* = 0.29; Additional file 1: Table S2). Stratifying patients into 5, rather than 2, subgroups based on mutational burden failed to convey improved predictive information (Additional file 3: Figure S2). Thus, while high mutational burden was associated with a slightly higher ORR amongst ICI-treated patients, the lack of association with OS casts doubts on the predictive value of mutational burden assessed as a standalone biomarker in clinical practice.

### Immune signature by unsupervised transcriptomics

**Fig. 2:**
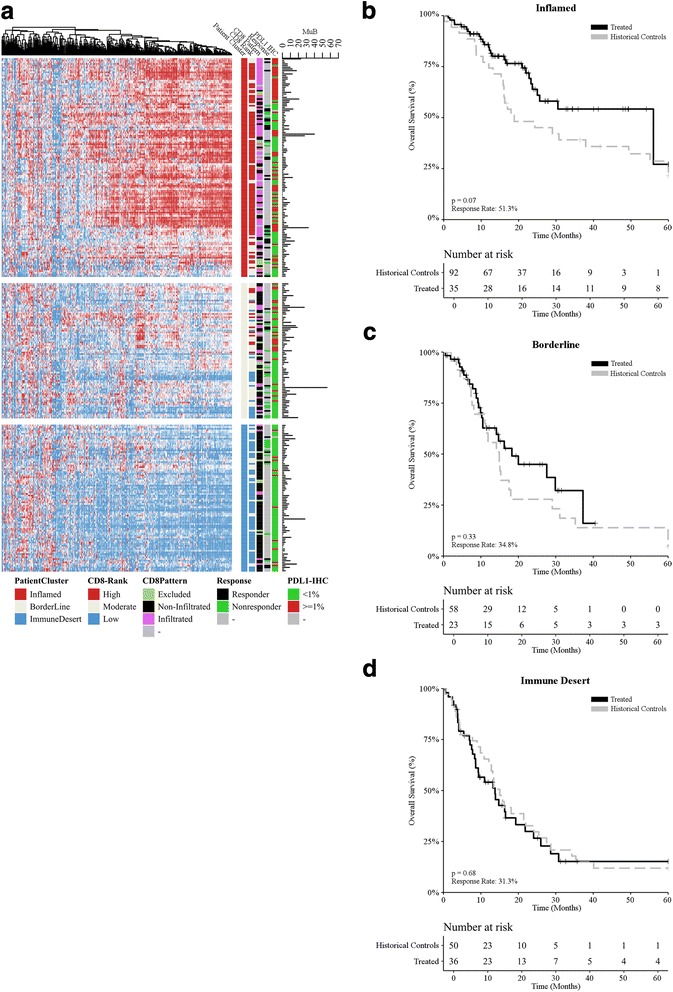
Immunological landscape of melanoma and its association with multiple variables. **a** Unsupervised hierarchical clustering (rows = patients, columns = genes) for 394 immune transcripts identified three major groups defined as “inflamed” (group 1), “borderline” (group 2), and “immune desert” (group 3). CD8 (assessed by RNA-seq) and PD-L1 expression levels (assessed by immunohistochemistry), response to ICIs (as per RECIST v.1.1), CD8 infiltration pattern (assessed by a trained pathologist, CM and APS), and mutational burden (assessed by whole-exon sequencing) are depicted. **b**-**d** Overall survival for melanoma patients from the (**b**) inflamed (*n* = 131), (**c**) borderline (*n* = 81), and (**d**) immune desert (*n* = 88) groups upon stratification based on treatment (historical controls versus ICI-based immunotherapy). *p* values are reported

Targeted transcriptomic analysis was performed on 394 immune transcripts including 11 housekeeping transcripts [17], covering 45 different gene functions, for 300 metastatic melanoma samples. Unsupervised hierarchical clustering identified three major groups of samples (Additional file 1: Table S2), with group 1 (*n* = 131; 44%) being the most common, and groups 2 (*n* = 81; 27%) and 3 (*n* = 88; 29%) of comparable frequencies (Fig. 2a). An analysis of gene functions at the individual gene level across the groups allowed for an immunological delineation of groups as: “inflamed” (group 1), “borderline” (group 2), and “immune desert” (group 3) (for detailed information, please refer to Additional file 1: Table S3). IHC for PD-L1 expression levels unveiled a statistically significant association (*p* = 1.63e-07) between PD-L1 positivity and the inflamed status (66/131; 50%), but PD-L1 positive tumors were still quite common amongst both the borderline (26/81; 32%) and immune desert (14/88; 20%) groups. Conversely, tumors with a high mutational burden were not overrepresented (*p* = 0·98) in the inflamed group (25/131; 19%) as compared to the borderline (22/81; 27%) and immune desert (16/88; 18%) groups. An overrepresentation test (v.test) showed that the inflamed group was enriched for tumors with a high number of CD8^+^ T cells, that the immune desert group was enriched for tumors with a very low number of CD8^+^ T cells, and that the borderline group contained a mixture of tumors with high or low CD8^+^ T-cell infiltration (for detailed information, please refer to Additional file 1: Tables S4 and S5). Objective responses to ICI-based immunotherapy, as assessed by RECIST v.1.1, were much more common amongst inflamed tumors (20/39; 51.28%), than amongst borderline (8/23; 34.78%) or immune desert (5/16; 31.25%) tumors (for detailed information, please refer to Additional file 1: Table S7). Altogether, these findings suggest that borderline tumors may be clinically closer to immune desert tumors than to inflamed tumors. In line with this notion, the inflamed status was associated with improved OS as compared with borderline or immune desert status amongst ICI-treated patients, but not amongst historical controls (Additional file 4: Figure S3). Moreover, amongst inflamed tumors receiving ICI-based immunotherapy there was a trend towards improved OS (Fig. 2b; *p* = 0.063; Additional file 1: Table S2). The same did not hold true for borderline (Fig. 2c; *p* = 0.33; Additional file 1: Table S2) and immune desert (Fig. 2d; *p* = 0.88; Additional file 1: Table S2) tumors.

**Fig. 3:**
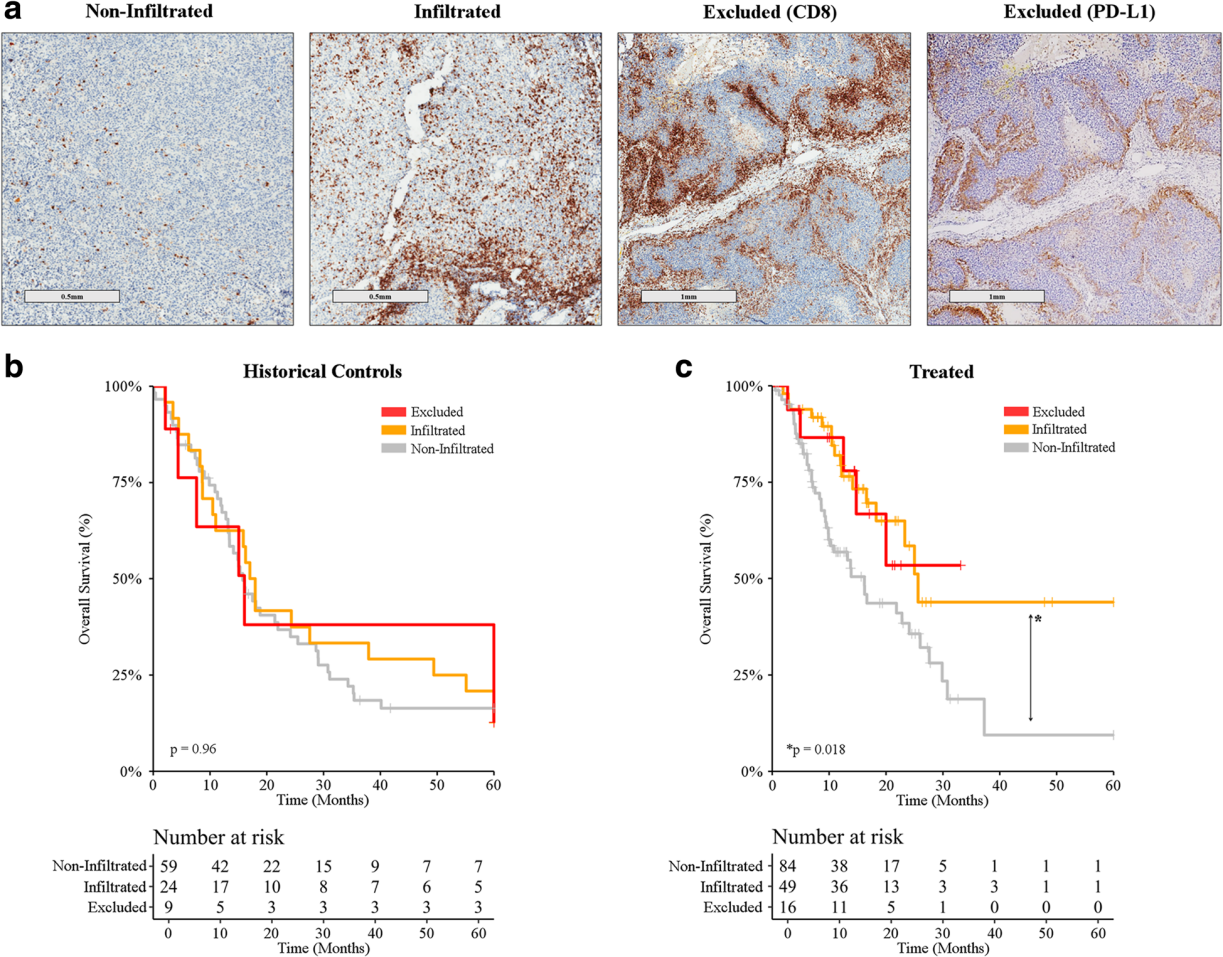
CD8^+^ T-cell infiltration pattern and clinical benefits from immune checkpoint inhibition. **a** CD8^+^ T-cell infiltration pattern was assessed by a trained pathologist upon immunohistochemistry with a CD8-specific antibody. Representative images are depicted (scale bar = 500 μm or 1 mm). **b**, **c** Overall survival upon stratification based on infiltration pattern (non-infiltrated, infiltrated, excluded) for metastatic melanoma patients treated (**b**) prior to the introduction of ICIs (historical controls; n = 94) and (**c**) ICI-treated melanoma patients (n = 137). For all comparisons *p* > 0.05

Since unsupervised sample clustering closely correlated with CD8^+^ T-cell quantification, a qualitative assessment of CD8^+^ T-cell infiltration pattern was performed by IHC (for detailed information, please refer to Additional file 7). Samples were then classified into 3 patterns: non-infiltrated, infiltrated, and excluded (Fig. 3a). Infiltration pattern correlated with immune group, with infiltrated tumors being mostly restricted to the inflamed group and non-infiltrated tumors being more common in the immune desert and borderline groups (Additional file 1: Table S2). Interestingly though, excluded tumors were evenly represented across all immune groups, representing about 10% of each. Infiltration pattern as assessed by IHC for CD8^+^ T-cells failed to identify patient subsets with an improved OS amongst historical controls (Fig. 3a; *p* > 0.96 Additional file 1: Table S3). Conversely, ICI-treated patients bearing infiltrated or excluded tumors before ICI treatment exhibited a superior OS as compared to patients with non-infiltrated tumors (Fig. 3b&c; *p* < 0.018 for all comparisons; Additional file 1: Table S2).

### Relationship between tumor genomics and the immune signature

Whole-exon sequencing of 409 cancer-related genes was performed with the intent of evaluating potential associations between specific mutations with immune group (inflamed, borderline, and immune desert) (Additional file 5: Figure S4; Additional file 1: Table S3). In particular, we harnessed the framework previously defined by the TCGA to examine whether immunological status and/or clinical response were associated with genetic driver subtypes: mutant BRAF, mutant RAS, mutant NF1, and triple WT. [16] From the 300 samples analyzed, a total of 264 samples (88%) exhibited at least one genomic alteration, CDKN2A loss (51%) being the most prevalent, followed by BRAF (38%), RAS (16%) and NF1 (7.3%) mutations (Additional file 5: Figure S4). Consistent with previous reports, 46% of tumors were classified as triple WT (Additional file 5: Figure S4). Tumors bearing BRAF, RAS or NF1 mutations were slightly overrepresented (60%; v.test = 1.71; *p* = 0.086) in the immune desert group. The loss of CDKN2A was also significantly associated (*p* = 0.00046) with the immune desert status, but not with OS (*p* > 0.05). Apart from RAS mutations, which were slightly associated with OS amongst historical controls (*p* = 0.02) but not ICI-treated patients (*p* = 0.28), no other statistically significant associations between genetic drivers of the disease and OS could be documented (data not shown).

### Predicting response to checkpoint blockade beyond PD-L1 levels and mutational burden

**Fig. 4:**
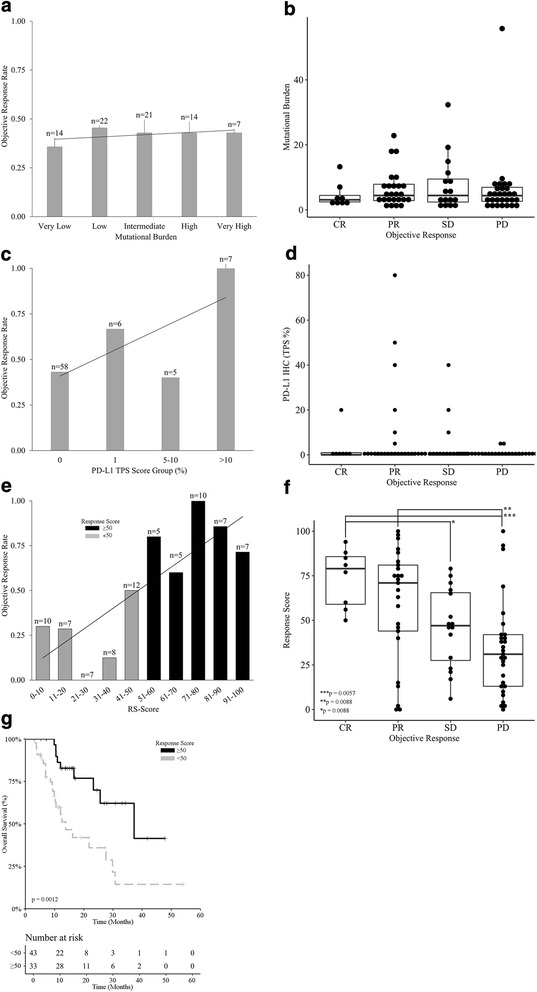
Predictive performance of the RS as compared to PD-L1 expression levels and mutational burden. **a** Response rates for mutational burden using a scale of very low, low, intermediate, high, and very high. While increasing response rates occur with an increase in mutational burden the range of values from 36% to 45% is also limited for clinical use. **b** Box plot shows a pair-wise comparison of mutational burden for CR, PR, SD, and PD. **c** Response rates for PD-L1 IHC using a tumor proportion score (TPS) with values of zero or negative, 1–4%, 5–10%, and > 10%. While increasing response rates occur with an increase in TPS the range of values from 40% to 100% is limited for clinical use. **d** Box plot shows a pair-wise comparison of PD-L1 IHC TPS for CR, PR, SD, and PD. **e** Objective response rates in groups of ICI-treated melanoma patients stratified by RS. Linear regression supports a dynamic range from 30% to 100%. **f** Box plot shows a pair-wise comparison of RS values for CR, PR, SD, and PD. **g** Overall survival upon stratification based on RS (≥ 50 versus < 50) for ICI-treated melanoma patients (n = 137), p value is indicated, for comprehensive immune profiling using response score (RS) with a bin width of 10. Response rate shows a dynamic range of values from near zero to greater than 95% with increasing RS. Survival curve for patients with a RS ≥ 50 and < 50 shows an improved survival for the former (*p* = 0.0012)

Transcriptomic data, mutational burden, and CD8^+^ T-cell infiltration pattern were combined to derive an algorithmic response score (RS) from a training set of 48 melanoma patients treated with ICI-based immunotherapy, and a validation cohort of 29 patients (Additional file 2: Figure S1 and Additional file 7 for more details). ORR of the combined training and test patients was 41.02% (8/78 CR, 24/78 PR, 16/78 SD, 29/78 PD) with the majority (68/78; 87.17%) treated with single agent ICI-based immunotherapy. Reference-normalized expression levels of 54 immune transcripts [17] co-expressed with PD-L1 were selected from a subset of 308 genes overexpressed in inflamed tumors (Wilcoxon rank sum test *p* < 0.05) and combined with mutational burden to derive a linear boundary of response versus non-response via machine learning (AUC > 0·95; Additional file 6: Figure S5). These values were then weighted based on infiltration pattern (as assessed by a trained pathologist) to obtain a score ranging from 0 to 100. RSs from the training and test cohorts were then combined to build a relative likelihood of response analysis upon linear regression fit (Fig. 4e; Additional file 1: Table S6). Patients experiencing a CR showed significantly higher RSs as compared to individual with SD (*p* = 0.0088) or PD (*p* = 0.0057). Similarly, patients with PR had a significantly higher RS than patients experiencing disease progression (*p* = 0.0088; Fig. 4f). The distribution of objective response rates from the training set versus response score groups was evaluated for predicting response resulting in threshold RS value of 50 (Additional file 1: Table S9). With this threshold on a combined dataset, ICI-treated patients with a RS ≥ 50 had a significantly improved OS and ORR of 82.9% as compared to 23.8% for patients with RS < 50 (Fig. 4g; *p* = 0.0012). Similar analyses on PD-L1 levels (Fig. 4a-b) and mutational burden (Fig. 4c-d) were performed for comparative purposes, showing no statistically significant impact (*p* > 0.05). These analyses demonstrated that the RS had a broad dynamic range for predicting response that spanned from 30% to 100%. In comparison, the assessment of PD-L1 levels by IHC and the evaluation of mutational burden by whole-exon sequencing had dynamic ranges spanning from 40 to 100% and 36–45%, respectively (Additional file 1: Table S7).

Sensitivity, specificity, positive predictive value (PPV), negative predictive value (NPV), and accuracy was evaluated for all biomarkers, including RS, PD-L1 IHC, and MuB for training, test, and combined datasets (Table 2). For the combined datasets RS demonstrated higher sensitivity (72.2%) compared to PD-L1 IHC (34.25%) and tumor mutational burden (32.5%), but with similar specificity (Table 2). Accuracy, defined as the ratio of correct to total predictions, was likewise higher for RS (80.52%) than for PD-L1 IHC (60.53%) and tumor mutational burden (55.13%). From a clinical standpoint, RS provides increased sensitivity and PPV while preserving specificity and NPV when compared to PD-L1 IHC and MuB, which is reflected in the improved accuracy. Similar results were observed for training and test sets (Table 2).

**Table 2:** Prediction performance for studied biomarkers

Prediction Method	Sensitivity	Specificity	PPV	NPV	Accuracy
Response Score (Training Dataset)	95.2%	74.1%	74.1%	95.2%	83.33%
Response Score (Test Dataset)	72.2%	81.8%	86.70%	64.30%	75.86%
Response Score (Combined)	84.6%	76.3%	78.60%	82.90%	80.52%
PD-L1 IHC (1% TPS)	34.2%	86.8%	72.20%	56.90%	60.53%
Mutational Burden	32.50%	78.90%	61.90%	52.60%	55.13%

## Discussion

Our data suggest that predicting the likelihood that melanoma patients will obtain durable clinical benefits from ICI-based immunotherapy by assessing PD-L1 expression levels or mutational burden has two major challenges. First, these biomarkers inform on key but limited components of the immune cycle [22]. Second, these parameters have only been used upon stratification towards binary (positive/negative) decision making that lacks dynamic range and suboptimal patient stratification for selection of therapy. In this study, non-ICI treated patients (historical controls) showed a limited survival advantage with positive PD-L1 expression (Fig. 1a), but no such effect was observed with mutational burden (Fig. 1c). In other tumor types, such as lung [23] and ovarian cancer [24] a high mutational burden has a survival advantage in non-ICI treated patients, but in both instances has been attributed to DNA-repair deficiency primarily via BRCA1, BRCA2, or POLE genes. In this study the mutational landscape of both non-ICI and ICI-treated cohorts was primarily driven by RAF/RAS mutations (Additional file 5: Figure S4) and might explain the lack of impact of mutational burden on survival in both instances.

Biomarkers that function as continuous variables, such as the algorithmic RS presented in this study, provide improved informational context in support of clinical decision making. The performance of the RS in this study is robust partially due to feature selection, which was independent of the training and validation set and initially derived from the unsupervised clustering of a reference population of mixed tumor histologies. Indeed, while the RS can be stratified based on a single cutoff value (as displayed in our survival analysis), the extended scope of inputs provides a much larger amount of information inherent to the numerical value of the RS, making it a suitable factor for improved decision making. Thus, the RS seems to convey more predictive value than the standalone assessment of PD-L1 levels or mutational burden. As most patients used to derive the RS in this study (87%) were treated with single agent ICI-based immunotherapy, our data suggest that a melanoma patient with a high RS should receive single agent (rather than dual agent) ICI-based immunotherapy in the clinical practice. Conversely, as most patients with a low RS failed to respond to single agent ICI-based immunotherapy, dual agent ICI-based immunotherapy would be the most appropriate choice for this group. Intermediate RS values should be evaluated on a per patient basis in the context of other parameters such as age, ECOG score, tolerance to side effects, etc.

While this work is not based upon a clinical trial it was a multi-institutional study with several important points. First, the features (genes) selected for the algorithmic analysis were chosen from a prior study [17]. Second, the algorithmic analysis was developed from patient samples from a single institution (RPCCC) and subsequently tested in a separate validation cohort from eight different institutions to mimic a real-world clinical scenario. Nonetheless, one of the major limitations of the present study is that our final training (48 patients) and test (29 patients) cohorts with RECIST v1.1 follow-up were relatively small. In addition, we operated with pooled data from patients receiving PD-1-targeting agents (pembrolizumab or nivolumab), CTLA4-targeting agents (ipilimumab), or both (nivolumab plus ipilimumab). Additionally, due to limited sample size in the test data set biomarker performance comparison for PD-L1 IHC, MuB, and RS was performed using the combined training and test data set which could potentially result in an over estimation of the accuracy of the comparative biomarker results. Our current dataset does not allow for the evaluation of these patients in a differential analysis with sufficient statistical power, therefore, a multi-institutional prospective trial will be undertaken to add confidence to our findings.

A potential confounding factor for this study was the use of clone 28–8 for PD-L1 IHC and the relevance of this stain for response to pembrolizumab as compared to its complementary diagnostic status for nivolumab. In this study the number of patients treated with pembrolizumab was greater than nivolumab and one could support that clone 22C3 would have been a better choice for this set of patients for PD-L1 IHC. While this was considered there is at least one multi-institutional study, the Blueprint PD-L1 IHC Assay Comparison Project, showing minimal differences between these two choices [25], albeit in another tumor type. Another potential confounding factor would be the role of driver mutations in context of mutational burden estimation and response to ICI. Prior meta-analyses in lung cancer have shown decreased response to ICI in EGFR or ALK mutant subgroups [26, 27], while BRAF mutations in melanoma have shown the opposite effect [15]. It is postulated that in some tumor types, such as lung cancer, these driver mutations do not contribute to “non-self” immunogenicity in a manner comparable to nonsynonymous passenger mutations [28]. In our estimation of tumor mutational burden, which is designed to filter ‘hot-spot’ variants, the effect of driver mutations and “non-self” immunogenicity therefore could not be the result of the observed lack of association between mutational burden and response.

Complex and multifactorial immune-biological mechanisms may not be easily captured by a more simplistic approach, such as one or a combination of single biomarkers. The algorithmic RS as presented in this study is a more complex approach that utilizes multiple factors that allows for the evaluation of a wide range of immunosuppressive and activating mechanisms that are not yet fully understood.

## Conclusions

In summary, we demonstrated that an algorithmic approach for a comprehensive evaluation of the mutational and immunological tumor landscape with a continuous (rather than dichotomous) response score provides superior informational context for predicting response to ICIs in metastatic melanoma.

## Additional files

Additional file 1: **Table S1** Clinical characteristics of the melanoma cohort. **Table S2** Survival analyses for studied biomarkers. **Table S3** Comprehensive gene expression and mutational profile of melanoma cohort. **Table S4** Over representation test results of categorical variables for gene expression clusters. **Table S5** Over representation test results of gene ranks for gene expression clusters. **Table S6** ORR for all biomarker groups studied. **Table S7** Objective response rates for studied biomarkers. **Table S8** Objective response counts for melanoma cohort. **Table S9** ORR for training set response score groups. (XLSX 1378 kb)

Additional file 2: **Figure S1.** Study schema. (TIF 18129 kb)

Additional file 3: **Figure S2.** Mutational burden and survival. (TIFF 33984 kb)

Additional file 4: **Figure S3.** Gene expression and survival. (TIFF 33984 kb)

Additional file 5: **Figure S4.** Genomic mutational landscape of melanoma cohort. (TIFF 16875 kb)

Additional file 6: **Figure S5.** Linear model AUC for “leave one out” validation of training set. (TIFF 16875 kb)

Additional file 7: Supplementary appendix including additional methods and results. (DOCX 40 kb)

## Abbreviations

AE: Adverse Events
CAP: College Of American Pathologists
CLIA: Clinical Laboratory Improvement Amendments
CR: Complete Response
CTLA4: Cytotoxic T-Lymphocyte Associated Protein 4
FDA: Food And Drug Administration
FFPE: Formalin-Fixed Paraffin-Embedded
ICI: Immune Checkpoint Inhibitor
IHC: Immunohistochemistry
IRB: Institutional Review Board
ORR: Overall Response Rate
OS: Overall Survival
PD: Progressive Disease
PD-1: Programmed Cell Death 1
PD-L1: Programmed Death-Ligand 1
PR: Partial Response
QC: Quality Control
RECIST: Response Evaluation Criteria In Solid Tumors
RS: Response Score
SD: Stable Disease
TCGA: The Cancer Genome Atlas
