# Supplementary figures and images for: Predicting response to checkpoint inhibitors in melanoma beyond PD-L1 and mutational burden
Source: J Immunother Cancer. 2018 May 9;6:32. doi: 10.1186/s40425-018-0344-8 (PMC5944039; doi:10.1186/s40425-018-0344-8)

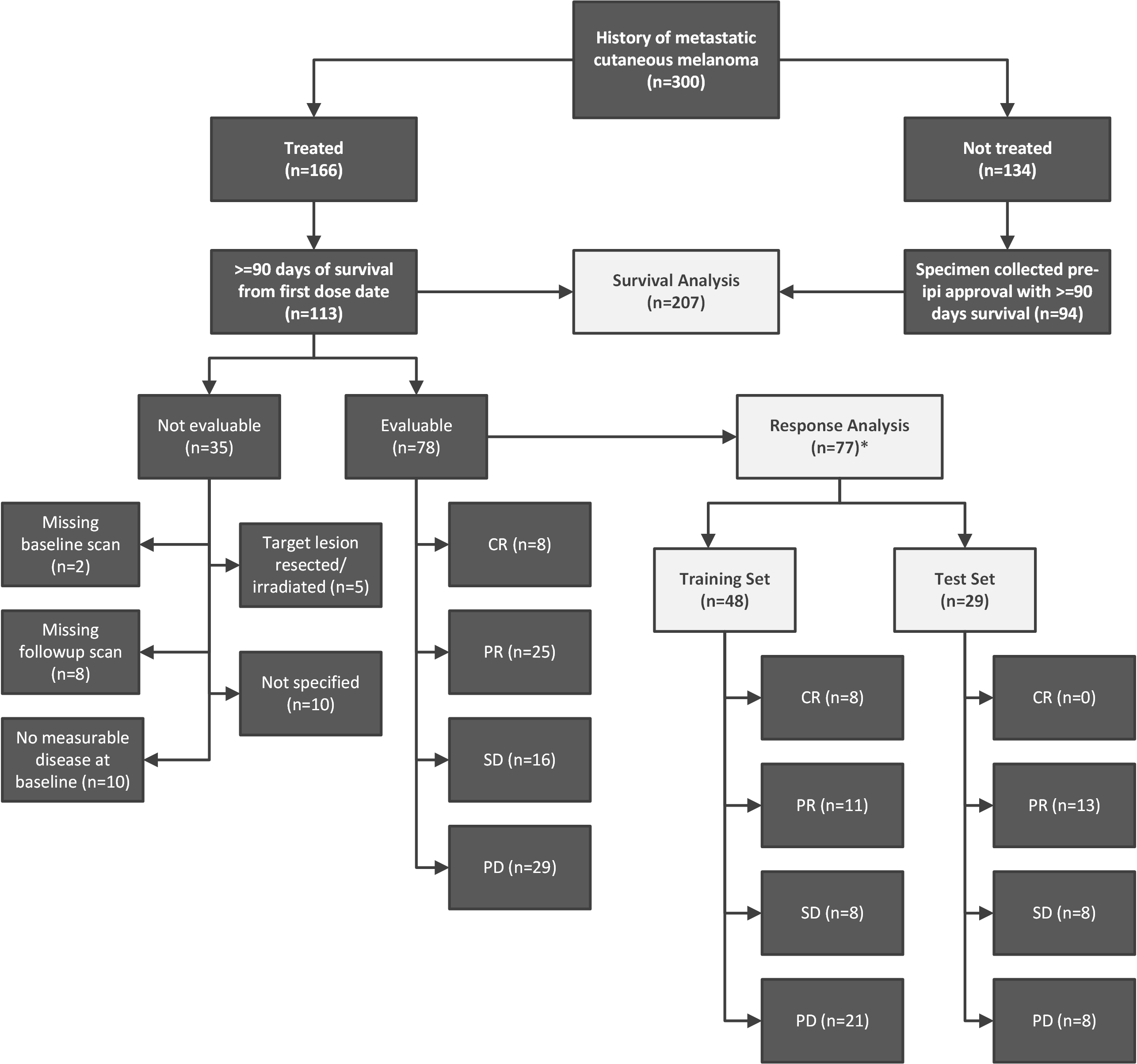

Supplement: Supplementary file 2 — Figure S1. Study schema. (TIF 18129 kb) [file 40425_2018_344_MOESM2_ESM.tif]

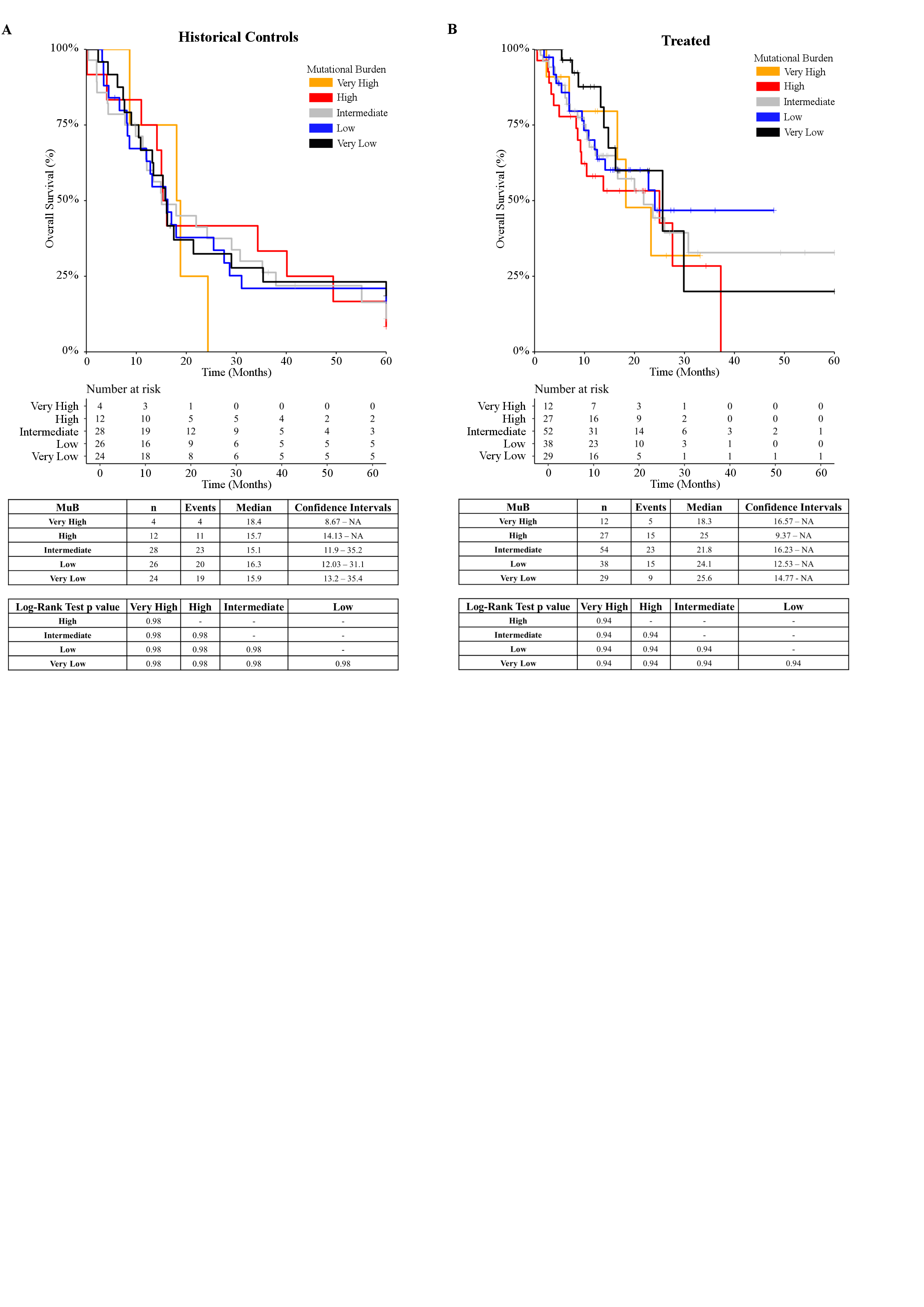

Supplement: Supplementary file 3 — Figure S2. Mutational burden and survival. (TIFF 33984 kb) [file 40425_2018_344_MOESM3_ESM.tiff]

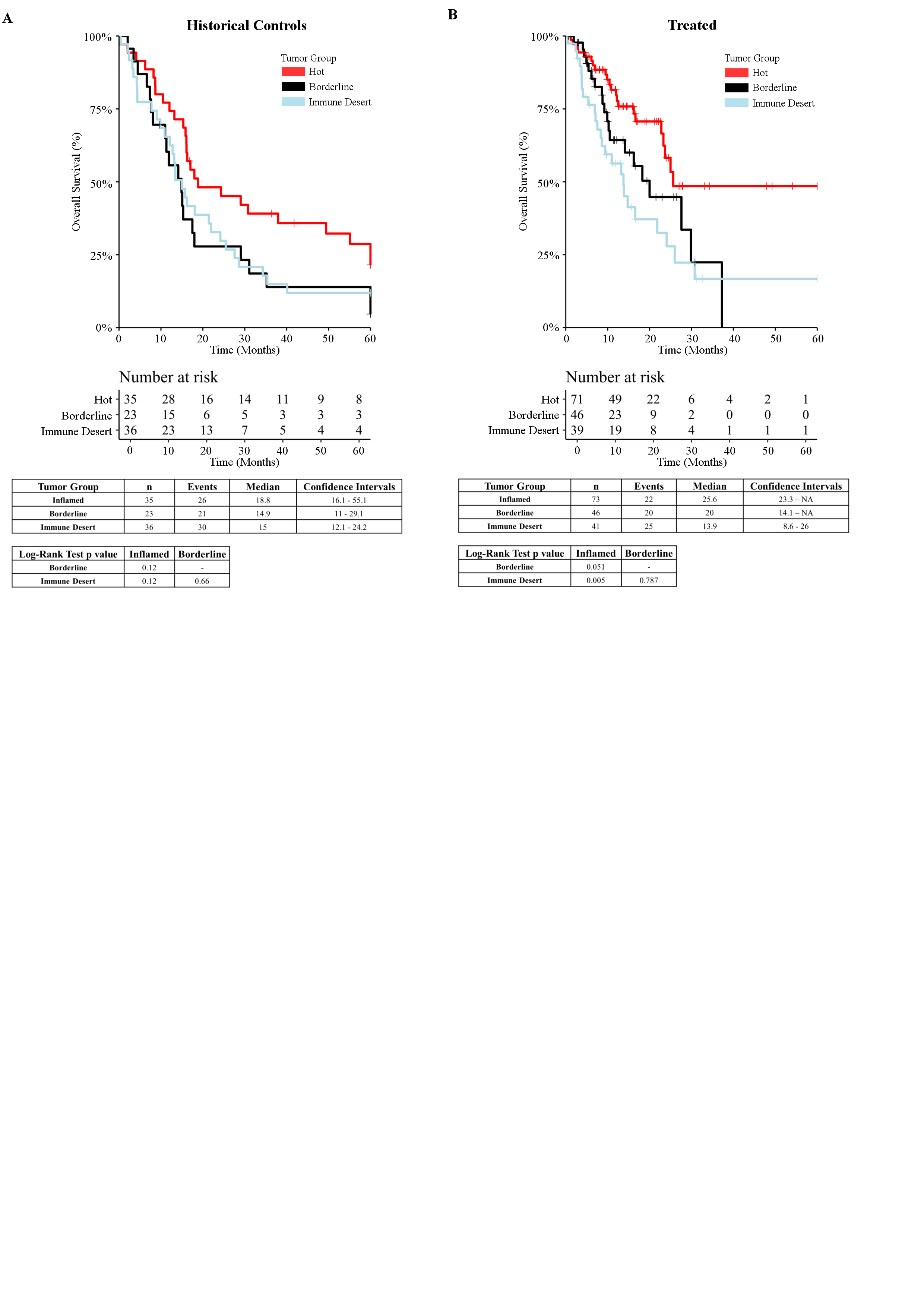

Supplement: Supplementary file 4 — Figure S3. Gene expression and survival. (TIFF 33984 kb) [file 40425_2018_344_MOESM4_ESM.tiff]

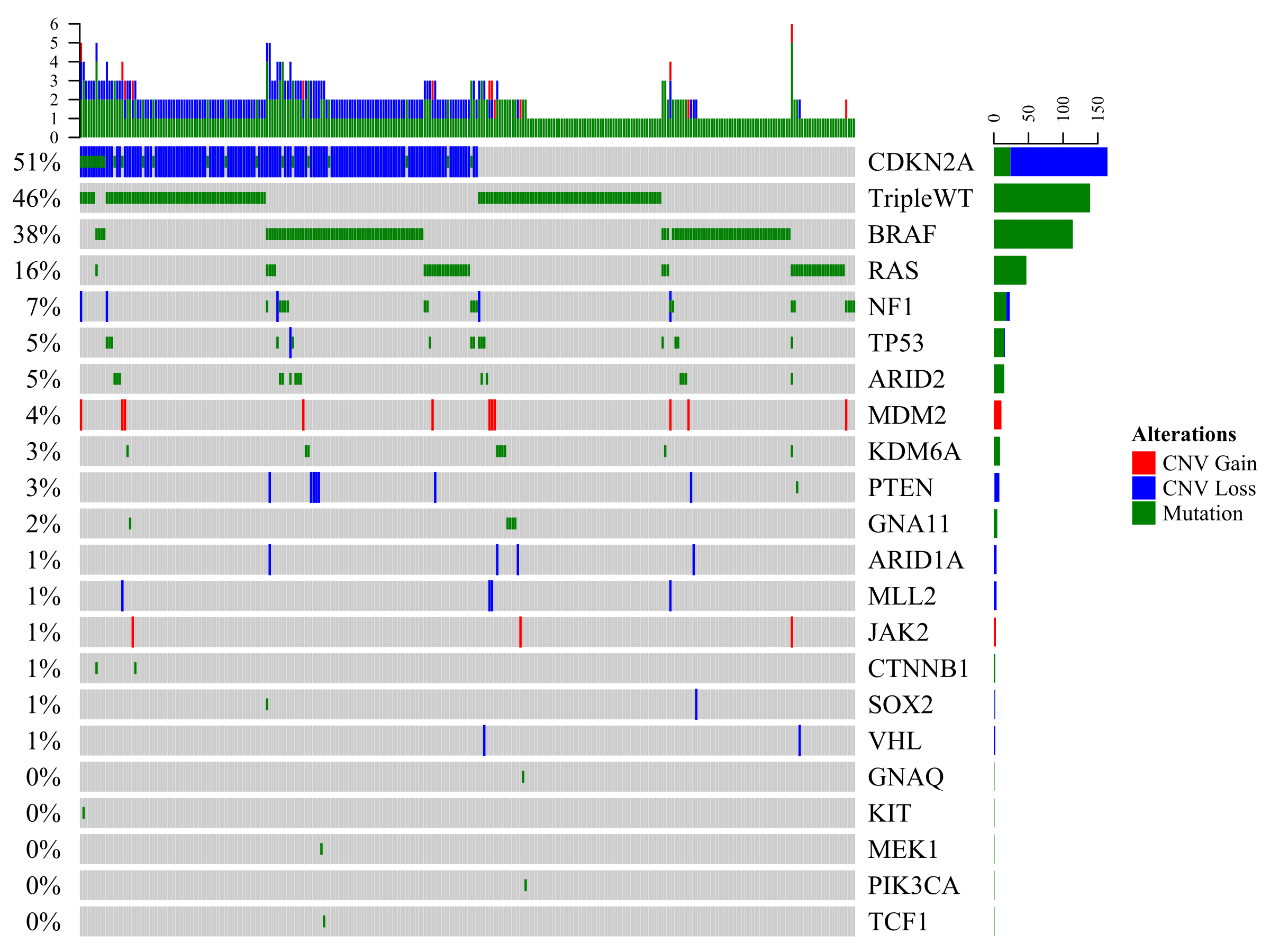

Supplement: Supplementary file 5 — Figure S4. Genomic mutational landscape of melanoma cohort. (TIFF 16875 kb) [file 40425_2018_344_MOESM5_ESM.tiff]

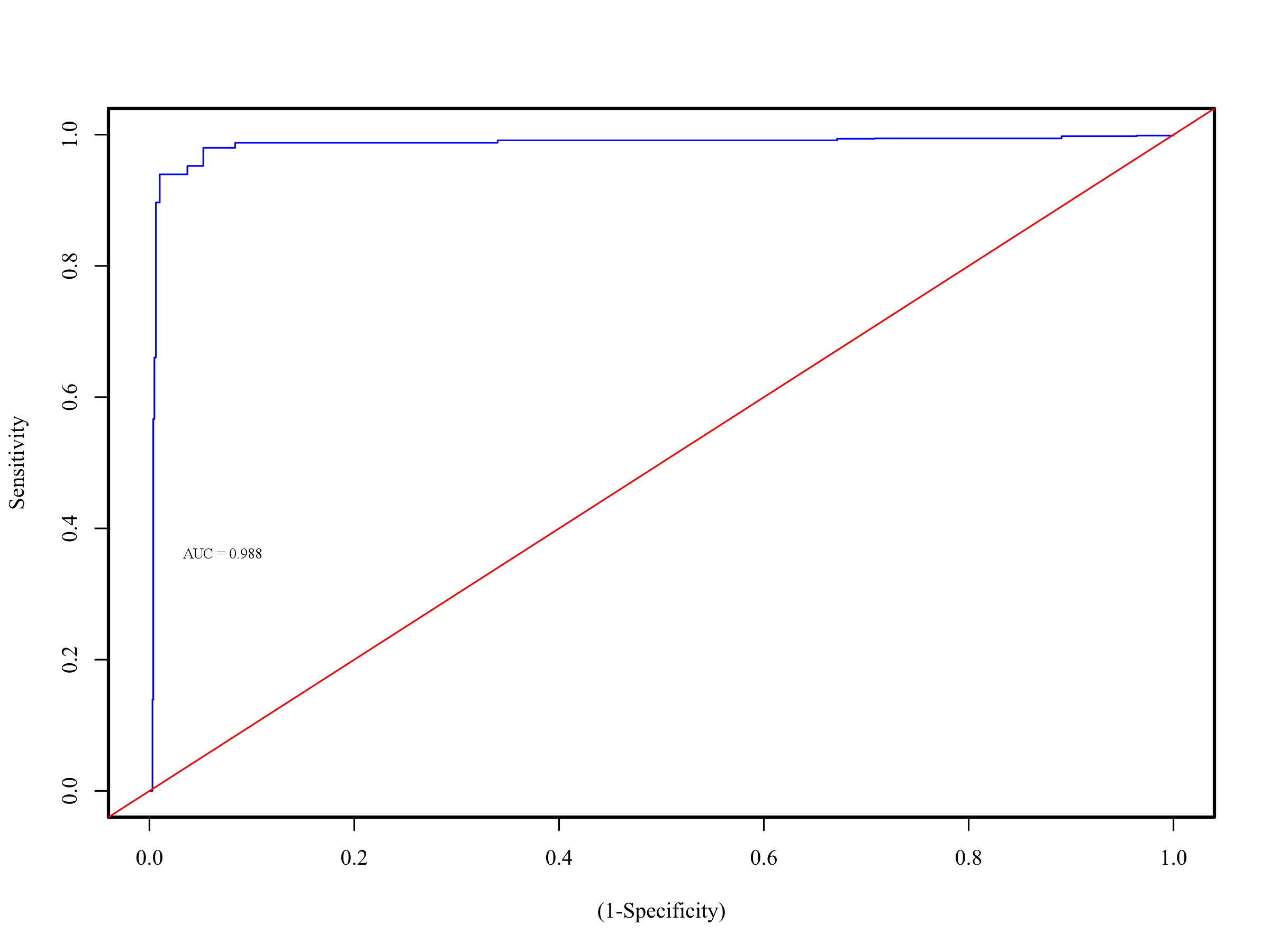

Supplement: Supplementary file 6 — Figure S5. Linear model AUC for “leave one out” validation of training set. (TIFF 16875 kb) [file 40425_2018_344_MOESM6_ESM.tiff]
