# Supplementary material for: Predicting response to checkpoint inhibitors in melanoma beyond PD-L1 and mutational burden
Source: J Immunother Cancer. 2018 May 9;6:32. doi: 10.1186/s40425-018-0344-8 (PMC5944039; doi:10.1186/s40425-018-0344-8)
Supplement: Supplementary file 7 — Supplementary appendix including additional methods and results. (DOCX 40 kb) [file 40425_2018_344_MOESM7_ESM.docx]

**Supplementary Material - Table of Contents**

**1) Supplementary Methods** p. 02

**2) Supplementary Figures – Uploaded Separately**

**3) Supplementary Figures Legends** p. 07

**4) Supplementary Tables – See Supplementary Excel Document**

**5) References** p. 08

**Supplementary Methods**

**Patient characteristics, clinical data and derived variables.** All eight (8) collaborating sites submitted complete basic patient characteristics and treatment information, including demographics (age at diagnosis, sex, race), last follow-up date, vital status (dead/alive) at last follow-up, date of death (when applicable), name of ICI, first and last dose dates, and date of specimen collection. All specimens from treated patients were collected prior to first dose of checkpoint blockade therapy. Six (6) sites also performed retrospective chart and imaging review to either provide objective response for patients with measurable disease at baseline, or determine when patients were not evaluable, following RECIST v1.1[1]. Qualifying baseline scans must have been performed no more than 45 days prior to first dose, and qualifying follow up scans must have been performed a minimum of 60 days post-first dose.

The variables listed below were derived from the provided data for analysis (See **Supplemental Table 1**) in the following manner: **ipi_approval:** dichotomous variable indicating whether the submitted specimen was collected prior to (pre) or after (post) initial approval of ipilimumab on March 25^th^, 2011, **clinical_benefit_12_mo:** dichotomous variable indicating whether patient was a complete responder (CR), partial responder (PR) or had stable disease (SD) with a minimum of 12 months of survival time from date of first dose (Responder) or if they had progressive disease (PD) or stable disease (SD) with less than 12 months of survival from date of first dose (non-responder), **follow_up_se_mo:** for all patients, the number of months from date of specimen collection (surgical event) to last date of follow up or date of death, and **followup_tx mo:** for treated patients, the number of months from date of first dose of checkpoint inhibitor to last date of follow up or date of death.

**Biospecimens and pathology review.** A board-certified anatomical pathologist (CM or APS) reviewed a hematoxylin and eosin (H&E)-stained tumor section to identify the region(s) to be tested. Tumor surface area on the H&E-stained section was ≥ 2mm^2^ per slide with tumor material presenting < 50% necrosis, at least 10% or 20% neoplastic nuclei (required for RNA-seq or DNA-seq, respectively). Regions identified by the pathologist were used as guides to scrape tissue from 3-5 unstained slides, each with one tissue section of 5µm thickness affixed. Genomic DNA and total RNA were simultaneously extracted by means of the Covaris truXTRAC FFPE RNA and DNA extraction kits (Covaris, Inc., Woburn, MA), as per manufacturer’s instructions with modifications. Post-extraction and purification, RNA and DNA were eluted in nuclease-free water, and yield was determined by the Quant-iT RNA HS Assay and Qubit dsDNA HS Assay Kits (Thermo Fisher Scientific, Waltham, MA), as per manufacturer’s recommendations. A predefined optimal yield of 10ng RNA and 30ng DNA was used as specimen acceptance criteria to ensure adequate library generation from each nucleic acid type, as per extensive analytical validation[2].

**Immunohistochemical assessment of PD-L1 and CD8.** The expression of PD-L1 on the surface of tumor cells was assessed in tumor samples by means of an automated immunohistochemical assay (28-8, Dako). A tumor was considered PD-L1 positive if ≥ 1% of viable tumor cells exhibited complete circumferential or partial linear plasma membrane staining at any intensity.

**Mutation and expression profiling.** The NGS assay utilizes the Oncomine Immune Response Research Assay (OIRRA) for gene expression profiling and the Comprehensive Cancer Panel (CCP) for mutational analysis (Thermo Fisher Scientific). Both these panels use multiplexed gene-specific primer pairs and NGS to amplify nucleic acids extracted from formalin-fixed paraffin-embedded (FFPE) slides. The complete OIRRA (covering 394 genes), as well as a subset adapted to quantify the expression of 54 validated target genes using 10 constitutively expressed housekeeping genes as normalizers, were utilized for analysis, whereas the CCP panel was employed to interrogate all 409 cancer-related genes for the estimation of mutational burden from genomic DNA, as previously described[2]. Furthermore, the CCP panel was used to identify key single-nucleotide variations (SNVs) and copy-number variations (CNVs). OIRRA and CCP libraries were prepared using the Ion AmpliSeq targeted sequencing technology (Thermo Fisher Scientific) followed by enrichment and template preparation using the Ion Chef system (Thermo Fisher Scientific) and sequencing performed on the Ion S5XL 540 chip. Sequencing data were first processed using the Torrent Suite software (v5.2.0) for reference mapping and base calling, during which previously-defined quality control (QC) specifications for mapped reads, on-target reads, mean read length, mean depth, uniformity, and percent valid reads were used as sample acceptance criteria[2].

RNA-seq absolute reads were generated using Torrent Suite’s plugin immuneResponseRNA (v5.2.0.0). Absolute reads were further normalized to yield normalized reads per million (nRPM) using previously described methods[2]. For all 394 genes, nRPM values were subsequently ranked (gene expression Rank) from 0 to 100 based on expression of these genes in a reference population of 167 samples representing a wide range of gene expression in various tumor types. CD8 expression rank was interpreted as low, moderate and high based on cutoffs of the 25^th^ and 75^th^ percentiles.

Mutation burden calling was conducted using Ion Torrent Suite software’s (v5.2.0) variantCaller (v5.2.0.34) plugin, which requires a minimal minor allele frequency (MAF) of 0.1 and a minimal coverage of 20X. In order to estimate mutational burden, a set of filters and annotations were applied to obtain a normalized count and interpretation of mutational burden qualified variants as previously described[2]. Briefly, these filters remove all synonymous variants, and non-synonymous variants ≥ 0.002 MAF in either 1,000 genomes project database, Exome Aggregation Consortium (ExAC) database, or Exome Sequencing Project (ESP) database. Mutational landscape for all the samples was calculated prior to the mutational burden filter application. To call SNVs, results from Ion Torrent Suite software’s (v5.2.0) variantCaller (v5.2.0.34) plugin were utilized. VEP v83 annotations of “deleterious” were used to call loss of function SNVs. Additionally, copy number gains and losses were estimated using read depth based approach. Ratio of target gene coverage to the average coverage of the target chromosome was used as a measure of CNV, wherein a coverage ratio ≥1.5 was called as a copy number gain and a ratio ≤ 0.5 as copy number loss.

**Statistical analyses.**

**Survival analyses:** For ICI-treated patients, survival was measured as time from date of first ICI treatment to death or last follow-up. In case of historical controls, survival was measured as time from date of date of biopsy proven Stage IV disease to death or last follow-up. Patients with less than 6 months follow-up time and vital status as alive were censored for this analysis. Five-year Kaplan-Meier survival curves were estimated using survfit function of Survival version 2.40.1[3] library in R v.3.3.1. To test for statistical difference between the survival curves, post-hoc log-rank test p-values (Benjamini-Hochberg corrected) were calculated using pairwise_survdiff function of survminer library version 0.4.0.

**Unsupervised clustering***.* Hierarchical clustering was performed on 394 gene expression ranks for all 300 samples using Pearson’s correlation as a measure of dissimilarity for both sample-wise and gene-wise clustering. Hierarchical tree was cut to reveal three distinct gene expression clusters named inflamed, borderline and immune desert. Over-representation test (v.test) was performed using catdes function of FactomineR[4] library in R. “v.test” is a statistical test to compare the mean of the gene expression rank in a cluster compared to the mean of gene expression rank of the gene in the overall population. For categorical variables, proportion of the variables is tested against the proportion of the variable in the overall population. In order to reveal clinical, mutational, and gene expression signatures characteristics for each cluster, “v.test” cutoff value ≥ 2 signified over-represented categories or genes whereas underrepresentation cutoff was ≤ -2.

**Response prediction***.* Training data set of 48 melanoma cases with RECIST v1.1 response data was used to train a generalized linear model with gene expression ranks and mutational burden. Area under the curve was estimated using leave one out validation on the training set. Default cutoff of 0 for linear model score was used. Model score was ranked from 0 to 100 for the training set. Model prediction score, CD8 expression levels and CD8^+^ T-cell infiltration pattern were weighed in to estimate the final response score for each sample. The resultant response score was tested on an independent validation set of 29 cases. Prediction performance metrics such as sensitivity, specificity for response score and other biomarkers was calculated independently on the training and test datasets, as well as in the pooled dataset. More specifically, the machine learning model uses 54 genes and mutational burden to calculate prediction score (PS). R library “glmnet” was used to fit a generalized linear model (logistic regression) with penalized maximum likelihood with the following parameters: family = binomial, lambda = 0.01 (minimum lambda achieved with cross validation in cv.glmnet function) and alpha = 0.2. Model was trained on 48 melanoma cases with RECIST v1.1 response. Below is a representative stepwise analytical workflow for RS calculation and response prediction:

**Step 1:**

For each sample S, calculate PS_(S)_ from the trained model.

**Step 2**:

For each sample S, calculate CD8 gene expression rank category CD8R_(S)_

- CD8R_(S)_ = Inflamed, if CD8 Rank ≥ 75
- CD8R_(S)_ = Borderline, if CD8 Rank > 25 and CD8 Rank < 75
- CD8R_(S)_ = Immune Desert, if CD8 Rank ≤ 25

**Step 3:**

For each sample S, calculate CD8 infiltration pattern CD8Pattern_(S)_

CD8Pattern_(S)_ could be one of the following

- Infiltrated
- Non-Infiltrated
- Excluded

**Step 4:**

For each sample S, calculate Response Score RS_(S)_

RS_(S)_ = W * PS_(S)_

- Where W is the weight based on CD8 Rank and infiltration pattern
  - If CD8R_(S)_ = Inflamed and CD8Pattern_(S)_ = Infiltrated, W = PS_(S)_
  - If CD8R_(S)_ = Borderline and [CD8Pattern_(S)_ = Infiltrated or Non-Infiltrated or Excluded], W = 1
  - If CD8R_(S)_ = Immune Desert and [CD8Pattern_(S)_ = Infiltrated or Non-Infiltrated or Excluded], W = 1
- For example:
  - RS_(S)_ for an inflamed and infiltrated sample: RS_(S)_ = PS_(S)_ * PS_(S)_
  - Whereas, RS_(S)_ for an Immune Desert and non-infiltrated sample: RS_(S)_ = 1 * PS_(S)_

**Step 5:**

Use linear regression equation to estimate response probability from a given RS_(S)_

Y = 0.01087(X) + 0.018059

Y = Response Probability

X = RS_(S)_

**Figure S1: Study schema.** Diagram illustrating classification of patients by treatment status, inclusion in survival analysis and inclusion in response analysis based on availability of imaging data for RECIST v1.1 assessment. Treated, received at least one dose of checkpoint inhibitor as next treatment following specimen collection; CR, Complete Response; PR, Partial Response; SD, Stable Disease; PD, Progressive Disease. *One case excluded from response analysis due to incomplete results for CD8 infiltration pattern by immunohistochemistry (IHC).

**Figure S2. Mutational burden and survival.** Melanoma patients were stratified into five mutational burden categories based on the number of non-synonymous variants detected: Very Low (black; < 1.775 mut/Mb), Low (blue; ≥ 1.775 to < 3.55 mut/Mb), Intermediate (gray; ≥ 3.55 to < 7.10 mut/Mb), High (red; ≥ 7.10 to < 14.20 mut/Mb) and Very High (yellow; ≥ 14.20 mut/Mb). **A.** Overall survival was not influenced (p > 0.05) by mutational burden in historical controls (n = 94), which are melanoma patients treated with surgery prior to the availability of immune checkpoint blockers (1992-2010). **B.** Similarly, there was no association between mutational burden and overall survival in patients (n = 160) treated with FDA-approved immune checkpoint blockers (n = 160).

**Figure S3. Gene expression and survival.** Melanoma patients were stratified into three immunological classes based on gene expression signature: Inflamed (red), Borderline (black) and Immune Desert (blue). **A.** Overall survival was not influenced (p > 0.05) by immunological class in historical controls (n = 94), which are melanoma patients treated with surgery prior to the availability of immune checkpoint blockers (1992-2010). **B.** Overall survival was significantly influence (p < 0.05) by immunological class in patients treated with FDA-approved immune checkpoint blockers (n = 160).

**Figure S4. Genomic mutational landscape of melanoma cohort**. Genes, mutation frequencies and subtypes of 300 biopsies from melanoma patients sequenced for somatic variations in 409 cancer related genes.

**Figure S5. Linear model AUC for “leave one out” validation of training set.** Receiver operating characteristics (ROC) with area under the curve (AUC) for “leave one out” validation of the linear model on the melanoma patient training set (n = 48) for 54-gene and mutational burden model.

**References**

1. Eisenhauer EA, Therasse P, Bogaerts J, Schwartz LH, Sargent D, Ford R, et al. New response evaluation criteria in solid tumours: Revised RECIST guideline (version 1.1). Eur J Cancer. 2009;45:228–47. doi:10.1016/j.ejca.2008.10.026.

2. Conroy JM, Pabla S, Glenn ST, Burgher B, Nesline M, Papanicolau-Sengos A, et al. Analytical Validation of a Next-Generation Sequencing Assay to Monitor Immune Responses in Solid Tumors. J Mol Diagnostics. 2018;20:95–109. doi:10.1016/j.jmoldx.2017.10.001.

3. Therneau TM, Lumley T. Survival Analysis Guide. Cran. 2017;:143. doi:10.1016/j.jhydrol.2011.07.022.

4. Lê S, Josse J, Husson F. FactoMineR : An R Package for Multivariate Analysis. J Stat Softw. 2008;25:1–18. doi:10.18637/jss.v025.i01.
